# The Intriguing Connections between von Willebrand Factor, ADAMTS13 and Cancer

**DOI:** 10.3390/healthcare10030557

**Published:** 2022-03-16

**Authors:** Chanukya K. Colonne, Emmanuel J. Favaloro, Leonardo Pasalic

**Affiliations:** 1 Department of Haematology, Institute of Clinical Pathology and Medical Research (ICPMR), NSW Health Pathology, Westmead Hospital, Sydney, NSW 2145, Australia; chanukya.colonne@health.nsw.gov.au (C.K.C.); or leonardo.pasalic@sydney.edu.au (L.P.); 2 Sydney Centres for Thrombosis and Haemostasis, Sydney, NSW 2145, Australia; 3 Faculty of Science and Health, Charles Sturt University, Wagga Wagga, NSW 2650, Australia; 4 Westmead Clinical School, University of Sydney, Westmead Hospital, Sydney, NSW 2145, Australia

**Keywords:** cancer, von Willebrand factor, ADAMTS13, thrombosis, metastasis, thrombotic thrombocytopenic purpura, acquired von Willebrand disease, bleeding, biomarkers

## Abstract

von Willebrand factor (VWF) is a complex and large protein that is cleaved by ADAMTS13 (a disintegrin and metalloproteinase with thrombospondin type 1 motif, member 13), and together they serve important roles in normal hemostasis. Malignancy can result in both a deficiency or excess of VWF, leading to aberrant hemostasis with either increased bleeding or thrombotic complications, as respectively seen with acquired von Willebrand syndrome and cancer-associated venous thromboembolism. There is emerging evidence to suggest VWF also plays a role in inflammation, angiogenesis and tumor biology, and it is likely that VWF promotes tumor metastasis. High VWF levels have been documented in a number of malignancies and in some cases correlate with more advanced disease and poor prognosis. Tumor cells can induce endothelial cells to release VWF and certain tumor cells have the capacity for de novo expression of VWF, leading to a proinflammatory microenvironment that is likely conducive to tumor progression, metastasis and micro-thrombosis. VWF can facilitate tumor cell adhesion to endothelial cells and aids with the recruitment of platelets into the tumor microenvironment, where tumor/platelet aggregates are able to form and facilitate hematogenous spread of cancer. As ADAMTS13 moderates VWF level and activity, it too is potentially involved in the pathophysiology of these events. VWF and ADAMTS13 have been explored as tumor biomarkers for the detection and prognostication of certain malignancies; however, the results are underdeveloped and so currently not utilized for clinical use. Further studies addressing the basic science mechanisms and real word epidemiology are required to better appreciate the intriguing connections between VWF, ADAMTS13 and malignancy. A better understanding of the role VWF and ADAMTS13 play in the promotion and inhibition of cancer and its metastasis will help direct further translational studies to aid with the development of novel cancer prognostic tools and treatment modalities.

## 1. Introduction

von Willebrand factor (VWF) is a complex and large multimeric glycoprotein that plays an essential role in regulating hemostasis [1]. VWF mediates both platelet adhesion to the subendothelial collagen matrix as well as platelet–platelet interactions under high shear conditions, thereby promoting the primary attachment of platelets to damaged endothelia and facilitating primary hemostasis [2]. The VWF is composed of a series of oligomers, each comprising of a variable number of subunits that range from a minimum of 2 subunits to a maximum of >40 subunits [1]. These oligomers are joined together to construct VWF multimers. The ability of VWF to maintain hemostasis is dependent upon its multimeric structure, with high molecular weight (HMW) multimers boasting greater hemostatic ability [3].

The VWF is synthesized in endothelial cells and megakaryocytes as proVWF subunits, that go on to dimerize in the cellular endoplasmic reticulum, and subsequently form multimers in the Golgi apparatus [1]. The VWF multimers are stored as large multimeric forms termed ‘ultra-large VWF’ (UL-VWF) in Weibel–Palade bodies (WPB) of endothelial cells and alpha granules of platelets [1]. Exocytosis of WPB and the release of VWF multimers are triggered by two main mechanisms, namely by agonists such as histamine and thrombin increasing the concentration of cytosolic Ca^2+^, and by agonists such as epinephrine and vasopressin acting via increasing cyclic adenosine monophosphophate levels [4].

VWF circulates in an inactive globular form upon initial release into circulation; however, it undergoes a conformational change when subjected to sufficient mechanical (‘sheer’) forces [5]. The mechanical force-dependent conformational change transforms VWF into an extended (‘elongated’) active form, thereby exposing critical domains involved in binding to platelets and collagen as well as the ADAMTS13 (a disintegrin and metalloproteinase with thrombospondin type 1 motif, member 13) cleavage site at the A2 domain [3] (Table 1). By cleaving these active UL-VWF multimers into smaller ones, ADAMTS13 regulates VWF multimer distribution and limits its prothrombotic action.

Reduced VWF antigen levels or reduced functionality of VWF results in a bleeding phenotype, with congenital defects or deficiencies leading to von Willebrand disease (VWD), the most common inherited bleeding disorder [6]. Alternatively, those with acquired defects or deficiencies of VWF are characterized as having acquired von Willebrand syndrome (AVWS).

As ADAMTS13 is responsible for moderating VWF size and function, a deficiency of ADAMTS13 can lead to increases in the VWF, in particular, HMW VWF forms. Severely deficient ADAMTS13 to less than a 10% level or activity results in the rare life-threatening disease of thrombotic thrombocytopenic purpura (TTP), in which an abnormal accumulation of HMW-VWF multimers results in greatly increased platelet binding and formation of thrombi that promote microvascular occlusion [7].

Thus, VWF and ADAMTS13 clearly have a central role to play in mediating hemostasis and thrombosis. Numerous reports of various malignancy-associated changes in VWF and ADAMTS13 levels have inspired this current review into exploring these documented changes and potential associations with bleeding and thrombotic phenomena as well as possible links to cancer metastasis and prognosis.

## 2. Acquired von Willebrand Syndrome and Cancer

AVWS is a rare bleeding disorder associated with similar clinical symptoms and laboratory features to VWD, where there is either a reduced activity or function of VWF, resulting in a bleeding diathesis [8,9,10,11]. In contrast to inherited VWD and in step with AVWS being an acquired disorder, there is a negative family history of bleeding. Cancer is one of the major underlying causes of AVWS, with hematological malignancies making up the majority of cases [12,13]. Indeed, lymphoproliferative disorders (LPD) are the most common cause of AVWS, representing approximately 50% of AVWS [13]. Myeloproliferative neoplasms (MPN) represent about 15% of AVWS, and solid tumors are more rarely associated, accounting for only about 5% of cases [12].

The pathophysiology of cancer associated AVWS is heterogenous with multiple different pathogenic mechanisms that ultimately results in increased VWF clearance from the plasma [9]. The increased VWF clearance can be via VWF/FVIII specific and non-specific autoantibody-mediated mechanisms, by adsorption of VWF on to malignant cell surfaces, and shear stress-mediated loss of HMWF VWF multimers [9,12]. The underlying mechanisms are not disease specific, and the same mechanism can cause AVWS in a variety of disorders [9,12]. A common mechanism in LPDs are autoantibodies directed against functional and non-functional VWF/FVIII domains as well as non-specific autoantibodies that form immune complexes with VWF and can result in increased VWF clearance via Fc-receptor mediated phagocytosis [9,14,15]. More rarely, the autoantibody can lead to an inhibitor phenomenon due to the antibody interfering with VWF function [16,17,18]. VWF can also undergo absorption onto malignant cell surfaces in LPD and MPNs, resulting in increased clearance of VWF from the plasma [19,20,21,22,23]. There is usually a preferential absorption of HMW VWF, resulting in a type 2 VWD-like pattern on laboratory assays [19,20]. High shear-stress conditions can also lead to AVWS, due to excessive cleavage of VWF by ADAMTS13 secondary to shear stress induced unfolding of VWF and selective loss of HMW VWF multimers, though this mechanism is generally more often associated with cardiac non-malignancy related AVWS [9,24].

Solid non-hematological malignancies are more rarely associated with AVWS, with about 5% of reported cases of AVWD in the literature [12]. Given the rarity, it is hard to estimate the true prevalence of individual neoplasms based on registry data, though the most commonly reported cases are in association with Wilms tumors, which are thought to secrete a plasma factor that increases VWF clearance [3,12,25,26].

The diagnosis of AVWS can be challenging and should be considered in patients with a bleeding diathesis with consistent laboratory tests and in patients with AVWS-associated disorders needing pre-operative planning [10]. Initial approaches to laboratory testing are similar to inherited VWD [6]. Inherited VWD is a complex clinical and laboratory diagnosis with frequent diagnostic inaccuracies due to the lack of a simple single diagnostic test, poor familiarity and understanding of the disease, significant pre-analytical, analytical and post-analytical issues, and the necessity to integrate the clinical and laboratory features to obtain an accurate diagnosis [6,27]. AVWD is no different and is likely commonly misdiagnosed or underdiagnosed, especially in patients that have never experienced a major hemostatic challenge such as major surgery or trauma [10,28,29].

In general, VWF screening studies may demonstrate a reduced or normal VWF:Ag (VWF antigen), FVIII (factor 8) activity, VWF:RCo (VWF ristocetin cofactor activity), and VWF:CB (VWF collagen-binding activity) [12,30]. These screening assays are particularly likely to be reduced in LPD-associated AVWS [10]. The qualitative platelet-related activity assays of VWF can often be lower than the quantitative VWF:Ag assay, with resultant reduced activity to antigen ratios often below 0.7, revealing a type 2A VWD-like pattern [11]. This is especially the case in MPN-related AVWS, where there is a selective loss of HMW-VWF multimers that is demonstrable on multimer electrophoresis studies, an assay available at specialized VWD testing laboratories [31,32,33]. Differentiating AVWS from inherited VWD can sometimes be challenging, but is an important distinction given the different treatment strategies [10]. The discovery of laboratory tests indicative of VWD, a lack of family history or long personal history of bleeding, and the presence of a condition known to be associated with AVWS should raise suspicion for an AVWS [8,10,11]. In difficult cases, testing for VWF-specific antibodies or inhibitors (to support a diagnosis of AVWS) and testing of family members and genetic analysis for VWD mutations (to instead support a diagnosis of inherited VWD) may be required. Though the yield of inhibitor testing is low, due to neutralizing antibodies being found only in a minority of patients, testing is recommended due to the associated severe bleeding phenotype and relative simplicity of testing by utilizing a Bethesda mixing study [10,11]. A markedly elevated VWF propeptide to VWF:Ag can help identify AVWS due to increased VWF clearance, though it is not specific for AVWS, and will also be similarly elevated with the inherited type 1 C (Vincenza) VWD [34]. This non-specificity along with the lack of a standardized tests and limited availability constrains routine use of VWF propeptide testing [34].

Consistent with its heterogenous pathophysiology, the clinical implications of AVWS are also varied, with a broad range of clinical severity and bleeding phenotypes [10,12,13]. The main symptoms of AVWS are mild to moderately severe mucocutaneus bleeding or excessive bleeding following trauma or surgical procedures [10].

Gauging the prevalence of AVWS in various malignancies and more importantly the prevalence of clinically significant bleeding is hampered by the lack of large-sized prospective studies and the relative rarity or under-recognition of this condition. One prospective study reports a 7.5% prevalence rate of AVWS in patients with LPDs, with a 45% rate of this subgroup being active bleeders [16]. Indeed, AVWS associated with LPDs generally have a higher risk of clinically significant bleeding, and a retrospective registry analysis classified 87% of patients with LPD-associated AVWS as being bleeders [12]. MPNs are another major category of underlying disorders, with a reported AVWS prevalence of 11–34% in this group of disorders, and with >50% demonstrating laboratory evidence of AVWS [9,16,35]. In contrast to LPDs, MPN-associated AVWS appears to be less commonly associated with a severe bleeding phenotype, with 14–48% being classified as bleeders [12,16]. The higher prevalence of AVWS without clinical bleeding in MPNs is also likely related to the increased testing for AVWS in patients with essential thrombocythemia with high platelet counts prior to aspirin prophylaxis initiation. Wilms tumor, the most common non-hematopoietic malignancy associated with AVWS, has a reported AVWS prevalence of 8% in a prospective pediatric study [25]. Bleeding is usually absent, though a small proportion may have mild bleeding, and there are reports of significant bleeding in rare cases [12,25,36].

Treatment of AVWS depends on the underlying etiology and the expected or present bleeding phenotype and is summarized in Table 2 [8,9,10,11]. The general principle is to address the underlying disorder whenever possible, though this is not always feasible. Treatment modalities include desmopressin, VWF-containing concentrates, antifibrinolytics such as tranexamic acid, high dose intravenous immunoglobulins (IVIG), plasmapheresis, corticosteroids, along with chemotherapy and cytoreductive therapies to address underlying malignancies.

## 3. Cancer-Related TTP and VWF/ADAMTS13

TTP is a rare and life threatening thrombotic microangiopathy (TMA), with an annual incidence of approximately one case per million people [56]. TTP is defined as a microangiopathic hemolytic anemia (MAHA) with a moderate or severe thrombocytopenia and associated organ dysfunction, including typical neurological and renal dysfunction as well as fevers. TTP is driven by a marked deficiency of ADAMTS-13 activity to <10% by either an acquired autoimmune phenomenon or by inherited mutations to the ADAMTS13 gene in combination with an additional trigger [7,57]. Severe ADAMTS13 deficiency results in reduced VWF cleavage and the accumulation of UL-VWF multimers, which promote the formation of platelet-rich thrombi in arterioles and capillaries that trigger a thrombotic microangiopathy [57].

Even rarer are reports of TTP directly related to malignancy. We found only one report with objective evidence of a laboratory ADATM13 deficiency proven TTP in malignancy—a case series of TMA in patients with monoclonal gammopathies where a few patients were classified as having TTP [58]. Care must be taken in interpreting reports of ‘malignancy-associated TTP’ in the literature, as these simply reflect cases of TMA with features similar to TTP, with no reported severe ADAMTS13 deficiency or with no ADAMTS13 testing being performed to clarify this specific diagnosis [59,60,61,62]. There are case reports of cancer-treatment related TTP following immune check point inhibitor treatment [63,64,65,66,67,68,69] and with multiple myeloma treatment with bortezomib [70] and lenalidomide [71,72].

## 4. VWF, ADAMTS13 and Cancer Metastasis Risk

Apart from its more established roles underpinning hemostasis, VWF has more recently sparked interest for its potential role in tumor biology, including inflammation, angiogenesis and metastasis [73,74,75]. VWF levels are significantly increased compared to healthy controls in a variety of both hematological and non-hematological solid-organ malignancies, including breast, colorectal, non-small cell lung cancer, gastric, prostate, and acute lymphoblastic leukemia [73]. There is also evidence from multiple tumor cell lines revealing their ability to direct endothelial activation and cause secretion of VWF from endothelial WPB [76,77,78,79,80,81,82]. This tumor-mediated VWF secretion is via tumor secretomes acting on neighboring endothelial cells, including inflammatory cytokines, matrix metalloproteinases, and pro-angiogenic mediators such as VEGF-A (vascular endothelial growth factor A) [76,77,78,79,80,81,82,83].

There is also evidence demonstrating that VWF is released from both tumor cells as well as the tumor microenvironment in certain cancer patients, in addition to the VWF released from the usual sources of endothelial cells and megakaryocytes. Non-endothelial-origin tumor cell lines, such as osteosarcoma, colorectal cancer, hepatocellular carcinoma (HCC), gastric cancer, and gliomas, have been shown to produce VWF [84,85,86,87,88]. VWF expression has also been found in histopathological samples of patients with osteosarcoma, HCC, gliomas, and gastric carcinomas [84,85,87,88,89]. Immunofluorescence and electron microscopy also reveal VWF in structures resembling WPB, or pseudo-WPB, though there are some distinctions from endothelial cell WPB [85]. These pseudo-WPB display a more homogenous staining pattern of the VWF compared to the punctate cytoplasmic granular staining seen with WPB in endothelial cells [90], and do not show the characteristic perinuclear pattern of pre-VWF in the endoplasmic reticulum seen in endothelial cells [91]. Furthermore pro-VWF is not detected in the osteosarcoma cell line lysate electrophoresis [85].

While it is unclear at this stage as to what level the tumor-derived VWF contribute to systemic plasma VWF levels, it is likely that local VWF secretion provides a survival advantage to the neoplastic process. In fact, tumor cells are also thought to create a microenvironment rich in VWF by inducing the localized deposition of the VWF. There are examples of colon carcinoma [92], gastric cancer [89] and lung adenocarcinoma [77] displaying high levels of VWF expression in the tumor stroma, which is thought to be mediated by either an increase in the tumor vasculature VWF gene expression [77] or due to the high density of exposed collagen and leaky vasculature in solid tumors, leading to an increased accumulation of VWF via binding to the collagen binding A3 domain of VWF [93].

Evidence to support the VWF promoting tumor metastasis comes from in vivo mouse experiments, which demonstrate that VWF inhibition results in significantly reduced metastasis in disseminating colon carcinoma [94], melanoma [76,94], Lewis-bladder cancer [94], gastric cancer [85] and thyroid cancer [95]. In vitro studies also demonstrate that there is reduced tumor migration when a tumor-derived VWF is inhibited in HCC [87].

VWF supports tumor metastasis via multiple postulated mechanisms, including promoting the tumor extravasation process by stimulating pro-inflammatory signaling and increasing vascular permeability, aiding tumor cell adhesion to endothelial cells and stimulating angiogenesis into the tumor microenvironment, as well as assisting with the carriage of tumor cells to distant sites [73,96].

VWF is an acute phase reactant, its levels increasing during infection and inflammation as well as being a marker for vascular dysfunction [97,98,99]. There is emerging evidence, however, for VWF being a mediator of inflammation rather than just a marker of it [99]. VWF appears to play a role in leukocyte recruitment [99], indirectly, via directing the biogenesis of WPB that also contain inflammatory mediators such as P-selectin that aid in leukocyte rolling in the early phases of inflammation [100], or directly, by binding leukocytes to VWF [101], or via recruiting and activating platelets via its GP1b receptor which in turn can increase vascular permeability and support leukocyte recruitment [102,103]. The role of VWF in mediating vascular permeability is shown in an intracranial model of a hemorrhage, where VWF infusion resulted in increased vascular permeability that was attenuated by anti-VWF antibodies [104], as well as a study demonstrating how a subendothelial VWF is inhibitory to claudin-5 expression, a tight junction protein expressed on endothelial cells [105]. Given malignancies, especially advanced malignancies, are states of heightened inflammation, it is likely that VWF plays a similar role in malignancy-associated inflammation.

There are a number of studies supporting the idea that VWF facilitates tumor adhesion to endothelial cells [85,87,88,89,106,107]. Evidence for positive regulation of angiogenesis by VWF comes mainly from ischemic models [108] and wound healing studies where VWF is shown to bind pro-angiogenic factors such as PDGF, VEGF and FGF [109]. There is indirect evidence for VWF-mediated angiogenesis in an ADAMTS13^−/−^ mouse model of melanoma, where increased intraluminal VWF deposition induced by melanoma directly correlated with increased tumor vessel density [96]. Moreover, a recent study demonstrates how the overexpression of VWF in breast cancer cells upregulates VEGF-A-related angiogenesis [110].

VWF is also postulated to be a mediator of the hematogenous spread of tumors by facilitating the formation of a ‘platelet-taxi’ or by interacting directly with tumor cells to promote metastasis [73]. In the former case, VWF appears to assist tumor adherence to platelets and is thought to allow the tumor cells to travel through the vasculature attached to platelets [111,112,113]. Blocking this VWF–platelet interaction results in reduced tumor cell interactions with platelets [111,112,114]. In the latter case, it is theorized that VWF may also contribute to certain tumor metastasis, independently of platelets, via integrin receptors and tumor-expressed pseudo-GPIb-alpha receptors that can bind the VWF [115,116,117].

VWF may also promote the transformation of tumor cells to facilitate metastasis as well. For instance, VWF released by endothelial cells, when co-cultured with osteosarcoma cells, induces the osteosarcoma cells to undergo epithelial mesenchymal transitioning, which is an essential step in tumor metastasis [118].

In step with VWF having a role in tumor metastasis, ADAMTS13 may also have a supportive role to play here. Building on the theory that VWF may help to create a ‘platelet-taxi’ for tumor cells, it can be envisioned that larger VWF multimers may increase platelet adhesion to cancer cells and further promote metastasis. Reduced ADAMTS13 levels may, therefore, increase the chance of tumor metastasis by increasing the availability of large VWF multimers. Consistent with this, there are reports of lower levels of ADAMTS13 in patients with disseminated and advanced stage cancer compared to a more localized disease [119,120,121]. There are, however, some conflicting results that have come to light in brain and prostate tumors, where ADAMTS13 levels were not found to be correlated to the stage of malignancy and metastasis [122]. Therefore, whilst helpful in promoting the VWF–tumor–platelet-taxi, it is likely that low ADAMTS13 levels per se are not the sole driver of this phenomenon. It is interesting to note that both the brain and prostate express high levels of tissue factor, another coagulation-related factor that is associated with cancer regulation, tumor growth and metastasis [123,124]. Perhaps these hemostatic factors work in concert to promote tumor metastasis, similar to how a multitude of different factors come together to result in normal hemostasis.

At a paradox to the evidence supporting a pro-metastatic role for VWF, there is evidence to suggest that VWF also plays a role in inhibiting tumor angiogenesis and promoting apoptosis [73,125]. VWF knockout mice have increased the pulmonary metastatic burden in mouse models of metastatic Lewis-lung carcinoma and melanoma [126]. It is theorized that VWF deficiency may result in the increased availability of platelet GPIba to promote cancer metastasis [114]. More recent evidence supports the idea that UL-VWF may be pro-apoptotic to certain cancer cell lines, including melanoma, breast, kidney, liver and lung [126,127]. However, tumor cells expressing high levels of ADAM28, a novel VWF cleavage protease, displayed resistance to this VWF-induced apoptosis in vivo [127]. While ADAMTS13 levels decrease in a range of disseminated cancers, ADAM28 expression is increased with advanced metastatic disease, likely due to a local tumor-induced expression of ADAM28 [120,127,128].

Given the raised VWF levels and above discussed associations with malignancy and metastasis, it is intriguing to speculate whether there is an increased or decreased risk of malignancy in conditions with known low or high VWF levels. Non-group O blood groups, which are well-known to correlate with higher VWF levels, are associated with a slight but significant increased risk of certain malignancies in a number of observational studies, including pancreatic, gastric, lung, liver and ovarian neoplasms [129,130,131,132,133]. Whilst the quality of the evidence behind these findings are generally weak due to the study designs, and the possibility of confounders cannot be dismissed, it is certainly consistent with the evidence for the positive regulatory role of VWF in malignancy. Data surrounding the risk of malignancy are limited in VWD; however, an interesting association between VWD type and cancer was reported in an Italian cohort study, where VWD type 2 was found to be significantly overrepresented in the cancer cohort compared to the total VWD population [134].

## 5. VWF and ADAMTS13 as Cancer Biomarkers

Another emerging concept is utilizing VWF and ADAMTS13 as predictive and prognostic biomarkers of cancer, given their altered levels in certain malignancies, as discussed in the previous section.

Both VWF and ADAMTS13 have been explored as potential biomarkers of cancer to aid in the early detection of colon cancer [135] and HCC [87,136,137]. VWF incorporated into a panel with several other protein and glycomic plasma markers showed promising results in the early detection of colon cancer, with high VWF levels correlating with cancer risk [135]. HCC, another frequently surveilled cancer in at risk populations, was shown to be associated with a high VWF antigen to ADAMTS13 activity ratio in cirrhotic patients [137]. The VWF antigen to ADAMTS13 activity ratio outperformed other tested biomarkers for HCC, such as alpha fetoprotein, VEGF, and des-y-carboxy prothrombin, was comparable to the multivariate biomarker of alpha-fetoprotein-L3%, and exclusively correlated with both the tumor volume and stage as well as plasma VWF levels [137]. In contrast to these findings, and demonstrating the need for further studies, ADAMTS13 activity was found to be significantly higher in those developing HCC in a separate study incorporating both chronic hepatitis and cirrhotic patients, and was independently associated with the risk of developing HCC [138].

The correlation and predictive value of VWF and ADAMTS13 in the prognostication of malignancies such as lung, ovarian, colorectal, glioblastoma, breast, and hematological malignancies have also provided mixed results. High levels of VWF antigen is associated with increased mortality at 12 and 36 months in ovarian cancer, along with higher D-dimer and KLK6 levels (a human tissue kallikrein) [139,140]. In colorectal cancer, a high plasma VWF concentration was identified as an independent prognostic factor by multivariate analysis, correlating with advanced disease and poor prognosis in patients with metastatic disease [141]. High plasma VWF levels were a better biomarker for poor survival than the well-known tumor marker CEA (carcinoembryonic antigen) which was not significantly changed in accordance with advanced stage and poor prognosis [141]. A more recent study evaluated this concept further and found that reduced ADAMTS13 activity and higher VWF antigen levels are associated with metastatic disease and reduced event-free survival, independent of age, gender, and stage-independent predictors of mortality in colorectal cancer [142]. However this study also demonstrated that both CEA and another well-known tumor marker CA19-9 as also being significantly associated with mortality and/or a thromboembolic event in colorectal cancer [142].

A retrospective data analysis in glioblastomas revealed that pre-operative high VWF levels were associated with a significantly worse post-tumor resection survival and were thought to relate to VWF effects on tumor angiogenesis [143,144]. High levels of VWF were also associated with a worse survival even in lower grade gliomas [145]. There is a positive correlation between VWF concentration and invasive breast carcinoma, with higher VWF to serum soluble E-selectin ratios being associated with poorly differentiated tumors [146]. However, contrary to this association, extremely high levels of VWF and a lower VWF/soluble E-selectin ratio was associated with better survival rates and lower relapse risk in the same patients [146]. An elevated VWF/ADAMTS13 ratio was shown to be independently associated with poor prognosis and mortality in patients with advanced non-small-cell lung cancer (NSCLC) [128]. The elevated VWF/ADAMTS13 ratio was also positively correlated with high D-dimer, fibrinogen and factor VIII levels [128]. Whether this is due to metastasis and tumor progression or sequalae of a hypercoagulable state resulting in thrombosis and subsequent reduced survival is unclear [128]. Somewhat contrary to these findings, a more recent (albeit low powered) study demonstrated that VWF upregulation may be associated with a favorable prognosis in lung adenocarcinoma [147].

With regards to hematological malignancies, there is evidence to suggest that ADAMTS13 levels are reduced in patients with acute lymphoblastic leukemia (ALL), and that lower levels are correlated with high-risk ALL and increased inflammation [148]. An Egyptian study has shown that children with ALL who had unfavorable outcomes had higher levels of VWF [149]. Waldenstrom’s macroglobulinemia is associated with high VWF levels compared to controls, and increased VWF levels were related to a higher International Prognostic Scoring System (IPSS) score and beta2-microglobulin levels [150]. Extremely high VWF levels correlated with very poor prognosis and were independent to other disease or patient characteristics [150]. High VWF levels were found to be associated with a lower than 5-year survival and worse outcomes after first-line therapy independently of the international scoring system in Waldenstrom’s macroglobulinemia [151]. A pilot study in myelodysplastic syndrome has shown that it is associated with lower basal levels of ADAMTS13 compared to healthy controls, with reduced levels correlating with the IPSS score and being inversely correlated with prognosis, overall survival, and poorer response to therapy [152].

VWF has also been studied as a potential biomarker for cancer treatment response outcomes. In HCC, ADAMTS13 and VWF have been shown to be potentially useful biomarkers for the prognosticating treatment response of sorafenib [153], hepatic artery infusional chemotherapy [154] and liver resection [155]. VWF has also been proposed as a biomarker that has a predictive value for an early three-month response in recurrent glioma to bevacizumab treatment, a neutralizing monoclonal antibody against a vascular endothelial growth factor ligand [156]. Baseline VWF levels, along with a number of other angiogenic biomarkers, correlated with progression free and overall survival in patients with metastatic colorectal cancer receiving cepecitabine, oxaliplatin, and bevacizumab [157].

Collectively, the evidence for VWF as a robust cancer biomarker, either alone or in combination with other markers, such as ADAMTS13, at present remain inconclusive. Contradictory results are perhaps influenced by the heterogeneity of the studies, retrospective designs, small study numbers, and uncontrolled confounders. VWF release as an acute phase reactant likely further complicates matters, and may prove problematic due to lack of specificity, especially in early screening tools. One need not look further than the significant preanalytical variability seen when performing VWF assays for VWD testing to envisage the potential issues when utilizing VWF as a screening biomarker [27]. Further studies to determine whether tumor-released VWF can be differentiated from an endothelial cell, and a platelet-derived VWF on peripheral blood sampling will likely improve the specificity of the VWF as a biomarker. It is already known that a platelet-derived VWF has a unique post-translational modification compared to their endothelial counterparts and exists as a hyposialyated glycoform, which is more resistant to proteolysis [158,159]. It is therefore possible that tumor-related VWFs have such underlying differences in their structure or biology that will allow such differentiation.

## 6. VWF as a Potential Target for Cancer Therapy

Given the emerging findings suggestive of the broad role VWF plays in mediating tumor metastasis, it is tempting to consider the possibility of targeting the VWF and its associated interactions in anti-neoplastic therapy.

Blocking GPIba, the part of the GPIb-IX-V receptor complex that binds platelets to VWF, affects the VWF-GPIba interaction and markedly inhibited platelet, tumor cells and endothelial cell interactions and pulmonary metastasis potential in an in vitro and mouse in vivo study of pulmonary metastases [114]. The monoclonal antibodies used to target GPIba did not result in thrombocytopenia or significant bleeding complications [114] and remain an exciting option for translational investigations.

Histone deacetylase (HDAC) along with GATA6 and nuclear transcription factor Y (NFY) promotes endothelial cell VWF gene promotor activation [160]. HDAC inhibitors are a known class of anti-tumor medications that promote cell apoptosis and cell cycle arrest [161]. A specific HDAC inhibitor, MS-275, has been shown to significantly reduce tumor growth, decrease VWF-positive blood vessels, decrease lung metastasis and reverse epithelial-mesenchymal transition in an in vivo murine model of breast cancer [162]. Given the relationship between HDAC and VWF gene expression, and the previously discussed mechanisms by which the VWF may promote tumor metastasis, it is possible that the effect of HDAC inhibitors in attenuating breast cancer metastasis may include the targeting of VWF expression [125].

The main barrier to utilizing VWF as a potential target for cancer therapy is the relative infancy of our understanding of how VWF and ADAMTS13 mechanistically affect malignancy and metastasis, and the heterogeneity and inconsistencies reported in the literature. A further issue is the potential risks of altering the VWF/ADAMTS13/endothelial cell axis, and the risk of triggering AVWS or TTP and their associated morbidity and mortality in these vulnerable patients. Perhaps tumor directed therapy, rather than systemic therapy, such that VWF/ADAMTS13 is only altered in the tumor microenvironment, may assist with mitigating this risk.

## 7. VWF, ADAMTS13 and VTE in Cancer

Cancer is associated with an increased risk of VTE compared to patients without cancer [163], and is associated with lower survival and poorer prognosis [164]. Thrombosis is a leading cause of death in cancer patients receiving chemotherapy, only second to the risk of death from the progression of the cancer itself [165]. Multiple factors contribute to this increased risk of VTE in cancer, including patient and tumor factors, increased inflammation, endothelial and platelet dysfunction, microparticle secretion, and tissue factor expression [73,166]. Additionally, high plasma VWF levels appear to be independent predictors of VTE in cancer patients [119,120,167,168], and this risk is largely mediated by increased endothelial VWF expression [169]. It is thought that the accumulation of UL-VWF and downregulated ADAMTS13 levels lead to VWF-platelet aggregates and fibrin deposition in the tumor vasculature resulting in a prothrombotic milieu in certain disseminated malignancies [96].

Separate to the increased risk of VTE and its associated morbidity and mortality, it is likely that the cancer coagulopathy itself may be accelerating tumor progression and the cancer metastasis risk [73]. Treatment of a transgenic mouse model of melanoma with tinzaparin, a specific low molecular weight heparin (LMWH), resulted in reduced tumor vascular accumulation of VWF, reduced tumor growth, angiogenesis and metastasis [76]. This effect may be related to the interaction between heparin and VWF via the A1 region resulting in impaired GPIba binding [170], as a similar effect was not seen when fondaparinux, a thrombin inhibitor, was utilized for anticoagulation purposes [96]. Furthermore, adjunctive anticoagulation with LMWH appears to prolong survival in cancer patients in multiple observational studies [171,172]. Further experiments dissecting the role heparins have on affecting VWF accumulation, cancer thrombotic and metastasis risk are required prior to applying this to routine patient care.

## 8. Conclusions

VWF is a highly complex molecule that has a role in both physiological and pathological hemostasis, as well as being a mediator of inflammation, angiogenesis and cancer biology. Along with ADAMTS13, VWF in excess or in deficiency can lead to cancer-associated thrombosis or bleeding. It is likely that VWF and ADAMTS13 promote tumor metastasis via interactions and effects on endothelial cells, platelets and the tumor microenvironment. At the present, there is a need for large prospective studies, as well as further basic science analysis, of how the VWF/ADAMTS13/endothelial cell axis affects malignancy. A better understanding of the role that VWF and ADAMTS13 play in the promotion and inhibition of cancer and its metastasis will help to direct further translational studies to aid with the development of novel cancer prognostic tools and treatment modalities.

## Figures and Tables

**Table 1:** VWF domains and functions.

VWF Domain	Key Features	Function
A1 domain	GPIb binding	Binding to platelets (GPIb); binding site for heparin, sulfated glycolipids, snake venom botrocetin, type IV collagen
A2 domain	Cryptic Tyr1605-Met1606 (ADAMTS13) cleavage site	ADAMTS13 cleavage site
A3 domain	Binding site for fibrillar collagens types I and III	Facilitates anchoring of platelets to exposed subendothelial matrix
C1 domain	RGD (Arg-Gly-Asp) tripeptide sequence	Binding site for platelets (integrin alphaIIb/beta3 (GPIIb/IIIa))
D’-D3 domain	FVIII binding site	Stabilizes FVIII in the circulation and prolongs FVIII half life

**Table 2:** Malignancies associated with acquired von Willebrand syndrome (AVWS).

Underlying Conditions	Examples and References	Pathogenesis of AVWS	Prevalence of AVWS in Disorder Group	Bleeding Risk	Treatment
LPDs (~50% of AVWS)	1. MGUS, multiple myeloma and Waldenstrom macroglobulinemia [14,15,16,17,18,23,37]. 2. Chronic lymphocytic leukemia [16,38,39]. 3. Hairy cell leukemia [40]. 4. Non Hodgkin lymphoma [22,41].	Autoantibody mediated clearance of VWF. Malignant clone cell surface adsorption and clearance of VWF. Inhibitors in rare cases.	7.5%	Associated with the most severe bleeding symptoms, 45–87% are active bleeders.	Desmopressin or VWF-containing concentrates usually provide short-lived improvements in VWF and are useful for acute bleeds [42]. Antifibrinolytics can be a useful adjunct for mucocutaneous bleeding [10]. High dose IVIG can be effective in patients with IgG autoantibodies or IgG paraproteins [10,42]. Not effective for IgM paraproteins and plasmapheresis may be required [42]. Long term remission can be achieved with use of directed immunochemotherapy for underlying malignancy [10]. MGUS is often untreated if asymptomatic, but if bleeding is an issue, malignancy-directed treatment can be difficult due to small size of plasma cell clone and resultant treatment toxicities [10].
MPNs (~15% of AVWS)	1. Essential thrombocythemia [16,21,43]. 2. Polycythemia vera [16,44]. 3. Primary myelofibrosis [45]. 4. Chronic myeloid leukemia [16,46].	Malignant clone cell surface adsorption and clearance of VWF. Inhibitors in rare cases.	Laboratory evidence of AVWS displayed in >50%	Of the sample, 14–48 % active bleeders. Most common significant bleeding site is gastrointestinal [47].	Subclinical (laboratory-only) AVWS does not require treatment. Manage acute bleeding or surgical requirements by ceasing aspirin/anticoagulation therapy, antifibrinolytic therapy, and use of desmopressin and/or VWF-containing concentrates [10,43]. Cytoreduction or platelet apheresis as emergency measures to reduce platelet count in bleeding. Long-term remission can be achieved with cytoreduction therapy [16].
Non-hematological neoplasia (~5% of AVWS)	1. Wilms tumors [25,26,36,48]. 2. Primitive neuroectodermal tumors [49]. 3. Bladder adenocarcinoma [50]. 4. Lung adenocarcinoma [51]. 5. Prostate cancer [52]. 6. Renal cell carcinoma [53]. 7. Adrenal cortical carcinoma [54] 8. Hepatocellular carcinoma [55]	Plasma factor that increases VWF clearance. Inhibitors in rare cases.	Sample sizes too small for meaningful estimate	Sample sizes too small for meaningful estimate.	Acute bleeding is usually mild and responsive to desmopressin and/or VWF-concentrates. Benefit also reported with high dose IVIG use [12]. Long-term remission with treatment of underlying malignancy is expected [9].

## Data Availability

Not applicable.
